# Introducing the Global Register of Introduced and Invasive Species

**DOI:** 10.1038/sdata.2017.202

**Published:** 2018-01-23

**Authors:** Shyama Pagad, Piero Genovesi, Lucilla Carnevali, Dmitry Schigel, Melodie A. McGeoch

**Affiliations:** 1 University of Auckland, Auckland, 1072 New Zealand; 2 IUCN SSC Invasive Species Specialist Group, 00144 Rome, Italy; 3 Institute for Environmental Protection and Research (ISPRA), 00144 Rome, Italy; 4 Global Biodiversity Information Facility (GBIF), Secretariat, Universitetsparken 15, Copenhagen Ø DK-2100, Denmark; 5 Monash University, School of Biological Sciences, Clayton, Victoria 3800, Australia

**Keywords:** Biodiversity, Policy, Invasive species

## Abstract

Harmonised, representative data on the state of biological invasions remain inadequate at country and global scales, particularly for taxa that affect biodiversity and ecosystems. Information is not readily available in a form suitable for policy and reporting. The Global Register of Introduced and Invasive Species (GRIIS) provides the first country-wise checklists of introduced (naturalised) and invasive species. GRIIS was conceived to provide a sustainable platform for information delivery to support national governments. We outline the rationale and methods underpinning GRIIS, to facilitate transparent, repeatable analysis and reporting. Twenty country checklists are presented as exemplars; GRIIS Checklists for close to all countries globally will be submitted through the same process shortly. Over 11000 species records are currently in the 20 country exemplars alone, with environmental impact evidence for just over 20% of these. GRIIS provides significant support for countries to identify and prioritise invasive alien species, and establishes national and global baselines. In future this will enable a global system for sustainable monitoring of trends in biological invasions that affect the environment.

## Background & Summary

The movement of species by humans beyond their historical geographic ranges, and the resulting negative impacts that some of these species have, remains amongst the key threats to biodiversity and ecosystems. Humans have been responsible for intentional and unintentional species introductions for centuries^1^ with growing appreciation over the last century of the unintended and sometimes severe consequences of biological invasion^2,3^. For example, 3.9% of the world’s vascular plant species have been introduced, established and naturalised outside their native ranges by humans^4^. Species populations, species richness, composition and ecosystem function are impacted by alien species at multiple spatial scales^5,6^.

Central to managing biological invasions is sound taxonomy and accurate identification of the species involved, information on the historical (native) and introduced ranges of species, and knowledge of realised or potential environmental impacts^7^ (see Table 1 for definitions of terms). This information is used to conduct risk assessments, inform policy to prevent the introduction and spread of alien species and to design surveillance, early detection and rapid response protocols. It is also used to monitor and report on the success of management interventions and policy targets.

Cross-border trade and transport is the principal driver of new species introductions, and knowing which species are where is therefore central to evaluating risk to biodiversity and ecosystems, identifying priority species and slowing the rate of new invasions^8^. The importance of such information is recognised by Parties to the Convention on Biological Diversity under Aichi Target 9 (i.e., By 2020, invasive alien species and pathways are identified and prioritized, priority species are controlled or eradicated and measures are in place to manage pathways to prevent their introduction and establishment), as well as by the Sustainable Development Goals (https://gsa.github.io/sdg-indicators/15-8-1).

Significant progress is being made with collating information on, and estimating, alien species distributions globally, e.g.,^9–11^. However, open access to information on the distributions of introduced species remains challenging, especially for countries wanting to use such data for the purpose of policy, management and reporting^12^. Reasons for this include, for example, (i) the dynamic nature of species distributions and data currency, (ii) data coverage, quality and accessibility are poor in several regions, and (iii) existing information platforms for alien and invasive species vary in purpose, quality and taxonomic and geographic coverage^13,14^. Several countries have developed effective local solutions to dealing with the management of introduced and invasive species data, e.g., the European Alien Species Information Network (EASIN); the Great Britain Non-Native Species Secretariat (NNSS); the Norwegian Biodiversity Information Centre (NBIC), and the alien and invasive species-related Databases of the Secretariat of Pacific Regional Environment Programme (SPREP). However, globally representative, comparable data on the presence of introduced and invasive species remain lacking, yet essential for the reasons outlined above^7^.

One of the solutions adopted by countries to facilitate the management of biological invasions is the generation and upkeep of national checklists of introduced and invasive species. These provide the simplest form of useful information on the distribution of alien species; essentially, they represent a presence record for each species listed at the scale of the country (or other geopolitical or land management units, such as islands or protected areas). While checklists are operationally useful, they are necessarily dynamic and the evidence underpinning them is fraught with multiple forms of uncertainty. These encompass lack of knowledge (especially on current distributions and position of range edges), species misidentification, context dependence (such as scale of impact) and linguistic uncertainty (vague use of terminology)^15^. While checklists of introduced and invasive species will never be complete or perfectly accurate, their value can be significantly improved by the adoption of a systematic, standard process of collating and updating them that is transparent and evidence-based^15^.

Here we present the Global Register of Introduced and Invasive Species (GRIIS), which is designed to provide such a harmonised, open source, multi-taxon database that includes verified information on the presence of introduced and invasive species for most of the world’s countries. GRIIS was conceived within the framework of the Global Invasive Alien Species Information Partnership (GIASI Partnership) of the Convention on Biological Diversity (CBD) to support national governments to make progress towards achieving Aichi Biodiversity Target 9. Decision XIII/13 adopted by the Conference of the Parties to the CBD encourages countries and the scientific community to invest resources into initiatives, such as GRIIS, to achieve Aichi Target 9 (CBD/COP/DEC/XIII/13 https://www.cbd.int/doc/decisions/cop-13/cop-13-dec-13-en.pdf).

**Figure 1:**
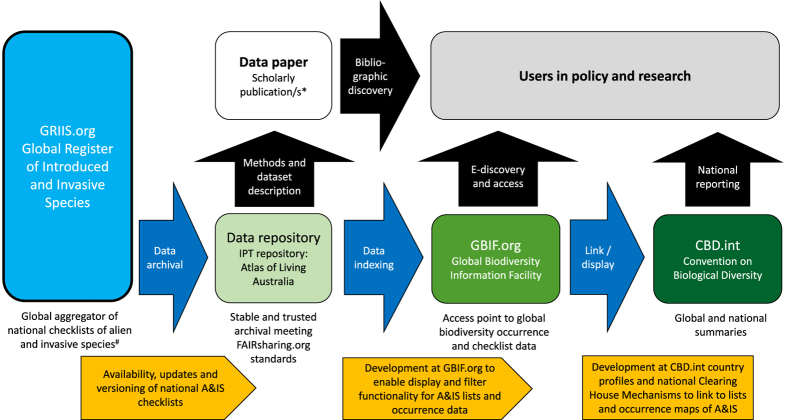
A schema for the role that the Global Register of Introduced and Invasive Species (GRIIS, left) is envisaged to play. Data from GRIIS feed into National Clearing House Mechanisms (CHMs) under the Convention on Biological Diversity (CBD) (right), via the Global Biodiversity Information Facility (GBIF.org) and its network of repositories. * This Scientific Data publication introduces GRIIS and provides the methods used to structure and populate it. # Country Editors contribute to verification and update of national checklists. The orange flags below outline the process by which information is updated and delivered. IPT; Integrated Publishing Toolkit. A&IS, alien and invasive species.

GRIIS has been developed to provide a sustainable basis for long term information delivery on alien (naturalised) and invasive species (Table 1) and to enable regular updates by countries, and forms a central part of a vision for global monitoring of biological invasions^7^. A key goal of GRIIS is to make country level GRIIS Checklists available to national governments. A road-map to achieve this has been envisioned using GBIF.org as the intermediary for the data standardization and information flow from GRIIS to science and policy users, such as the Parties to the CBD, worldwide (Fig. 1). Here we present 20 country Checklists (including three sub-lists of islands associated with specific country mainlands) as exemplars of the over 200 GRIIS Checklists to be made available and of the data available via the GRIIS Homepage. We use them here to describe the design, methods and use of the Global Register of Introduced and Invasive Species that includes data for 198 countries.

## Methods

### Species inclusion and designation of evidence of impact

GRIIS is populated by the Project Team (co-authors S.P., P.G, and L.C.) of the IUCN Species Survival Commission Invasive Species Specialist Group (ISSG), following a defined series of steps, including a systematic decision process to assign status and categories to the data where necessary. The intent is to ensure transparency and repeatability of the process as GRIIS is expanded and updated. This process is described below.

**Figure 2:**
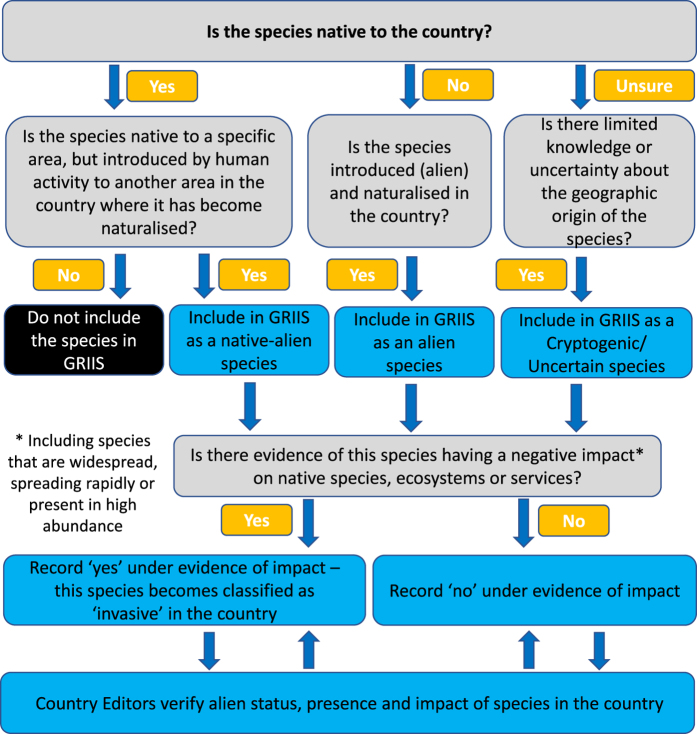
Systematic decision process and criteria for the inclusion of species in GRIIS for each country, as well as the designation of each species as having evidence of impact, or not, at a country scale. Note that ‘no evidence of impact’ does not mean that a species does not have any impact, rather that there is currently no evidence that it does.

A systematic decision-making process^15^ was adopted for (1) the inclusion of species in GRIIS via their designation as alien at a country level, and (2) the existence of evidence of impact for each species in each country (Fig. 2). The outcome of the first stage of this process was the assignment of origin and alien status (see Fig. 2, Table 2). Those species added to GRIIS were then assessed for evidence of impact (Fig. 2). Two forms of information were considered to qualify as evidence of impact on the environment:

1. When any authoritative source (e.g., from the primary literature or unpublished reports from country/species experts), described an environmental impact, and/or
2. When any source commented on the species as widespread, spreading rapidly or present in high abundance (based on the assumption that cover, abundance, high rates of population growth or spread are positively correlated with impact^13^).

At a country level therefore, each species ends up being designated as having, or not having, evidence of impact (Fig. 2). Across the full GRIIS dataset it is therefore possible to determine which alien (native-alien or cryptogenic/uncertain) species, and the proportion of species, for which there is evidence of impact in any country. GRIIS does not assess the severity of impact, volume of information providing evidence of impact or strength of evidence for a particular impact. Rather, GRIIS provides access to the sources providing the evidence. In this way the evidence can be further evaluated as needed for particular applications that could conceivably have a very broad range of contexts and objectives.

For example, in future it is envisaged that the magnitude of impact and confidence associated with assessing impact will be harmonised using the Environmental Impact Classification of Alien Taxa (EICAT)^16,17^. EICAT is a ‘system for classifying invasive alien species based on the nature and magnitude of their impacts’, supported by an IUCN members resolution in 2016 (https://www.iucn.org/theme/species/our-work/invasive-species/eicat). GRIIS will provide the foundation for input to country and global impact classifications via EICAT. GRIIS and EICAT provide significant opportunity for smooth integration between the delivery of evidence based lists to be assessed (GRIIS) and the assignment of ‘magnitude of impact’ categories to these species following the EICAT process^16,17^. All species listed as having evidence of impact in GRIIS would, once classified, fall within one of the five magnitude of impact categories in EICAT, and the species in GRIIS listed as having ‘no evidence of impact’ would be data deficient according to EICAT.

### Data collation and entry

Templates were used for the initial collation and recording of data and the preparation of draft checklists. All templates were accompanied by a set of ‘look-up tables’ with field options that describe the categories for the different information fields (Table 2). Draft checklists were compiled by collating information from a comprehensive literature and data search. To standardize the literature review as far as possible across countries, the following search protocol was followed. Biological Abstracts, BIOSIS, BioOne, Scopus and Google Scholar were searched using the following keyword combinations: Country name; alien species; introduced species; invasive species; exotic species; inventory; checklist; impacts. The dates of searches were recorded and no temporal limits were placed on searches. All abstracts and publications were screened to determine if they provided relevant lists of alien or invasive species.

The only criterion for considering the quality of evidence was that the source was authoritative and traceable. Source details are provided in all instances (Table 2). Governments, researchers and practitioners likely to have access to any additional, unpublished information and results of alien species surveys conducted in the country were contacted. Species records were compiled including all relevant annotations and any comments/remarks. Overall therefore, the information sources consulted included peer-reviewed scientific publications, national checklists and databases, reports containing results of surveys of alien and invasive species, general reports (including unpublished government reports), and datasets held by researchers and practitioners. Full citation and source information of records have been maintained for each country. In all cases the primary or secondary source of information for each species, country and record is recorded (Table 2, ‘Source’).

### Taxonomic harmonization and normalization

The GBIF.org Backbone Taxonomy^18^ was used to harmonise the taxonomy of species included in GRIIS. To harmonise names across countries, species lists were subjected to a process in which taxon rank and taxonomic status were identified and assigned (Table 2). Spelling and other errors in assigning species authorship were corrected at this stage.

### Data verification

Each draft checklist was submitted to a network of relevant Country Editors for review of both accuracy of records, and identification of significant gaps in the data for which the Editors have evidence. Data verification is an iterative process and the activity is declared complete on agreement by the Country Editors (see Data Citation 1 to Data Citation 23). Requests were sent to a wide network of Country Editors as part of this verification process of the country draft checklist; Country Editors included academics specialising in invasion biology and with good knowledge of alien and invasive species in that country, practitioners, and officials from government departments whose area of work included alien and invasive species. Given the inclusion of the wide range of taxonomic groups in GRIIS, as well as different environments, multiple editors were usually required. These Country Editors are authors of the published checklists (see Data Citation 1 to Data Citation 23).

Country Editors were given four months for the completion of the first iteration of the review process. Once comments were received, including new information sources, the new data were incorporated and species records revised as needed. The revised draft checklists were then returned to Country Editors for review. Depending on the composition of the editorial team for every country, interactions with editors were dedicated to the taxonomic or system specific sub- checklist they were working on. A minimum of four iterations have to-date occurred with every Editor. The GRIIS Checklist for a country was then associated with a final date and DOI-associated version on the agreement of both the Country Editors and the Project Team. As outlined above, annual major updates and a mid-year update of incremental additions and revisions are planned by the ISSG.

### Taxonomic, geographic and temporal coverage

Taxa in the following Kingdoms are represented in GRIIS: Animalia, Bacteria, Chromista, Fungi, Plantae, Protista (Protozoa), as well as Viruses. Environments include terrestrial, freshwater, brackish and marine. Taxa included in GRIIS are those that are introduced and have become naturalized and, in some countries, that also impact the environment, specifically biodiversity and ecosystems. Agricultural and socio-economic pests are excluded, except where they also have an environmental impact (as is often the case^19^). Species occasionally present, present only in containment facilities, extirpated or eradicated after a management intervention are not included in the lists.

GRIIS has global coverage, including the European Overseas Territories and Regions and currently includes data on 198 countries (with the process complete to the stage prior to country editor verification, i.e. excluding only the final two rows in Fig. 2). Where appropriate, sub-lists have been created for Oceanic Islands associated with particular country mainlands. The geographic coverage of each country encompasses the area within United Nations country borders. GRIIS Checklists for 20 countries (including three island sub-checklists) are included with this Data Descriptor as exemplars, and are available through GBIF.org. For the exemplar countries provided with this Data Descriptor, the sub-lists include Soqotra of Yemen and the Juan Fernandez Islands and Rapa Nui -(Easter Islands) of Chile.

Records included in GRIIS encompass the earliest documented records found during the search process to the most recent accessed record prior to the date of the latest version of GRIIS. Updates, data integrations and data revisions, requested by Country Editors or communicated through the contact page of GRIIS, will be screened in consultation with Country Editors. It is envisaged that the network of Country Editors will provide updates on new alien species records, as well as evidence of impacts and any changes in the status of alien and invasive species in their countries. The intention is for ongoing, regular consultation with the network of Country Editors to validate and verify any new data and information. All incremental and annual updates and revisions will be implemented as described. A version number is assigned to every version of the GRIIS Checklists at the conclusion of an annual update (for e.g., ver1, ver2). All incremental updates within a calendar year will result in a subsidiary number being assigned to the current version (e.g., ver2.1, ver3.2).

### Data summary

**Figure 3:**
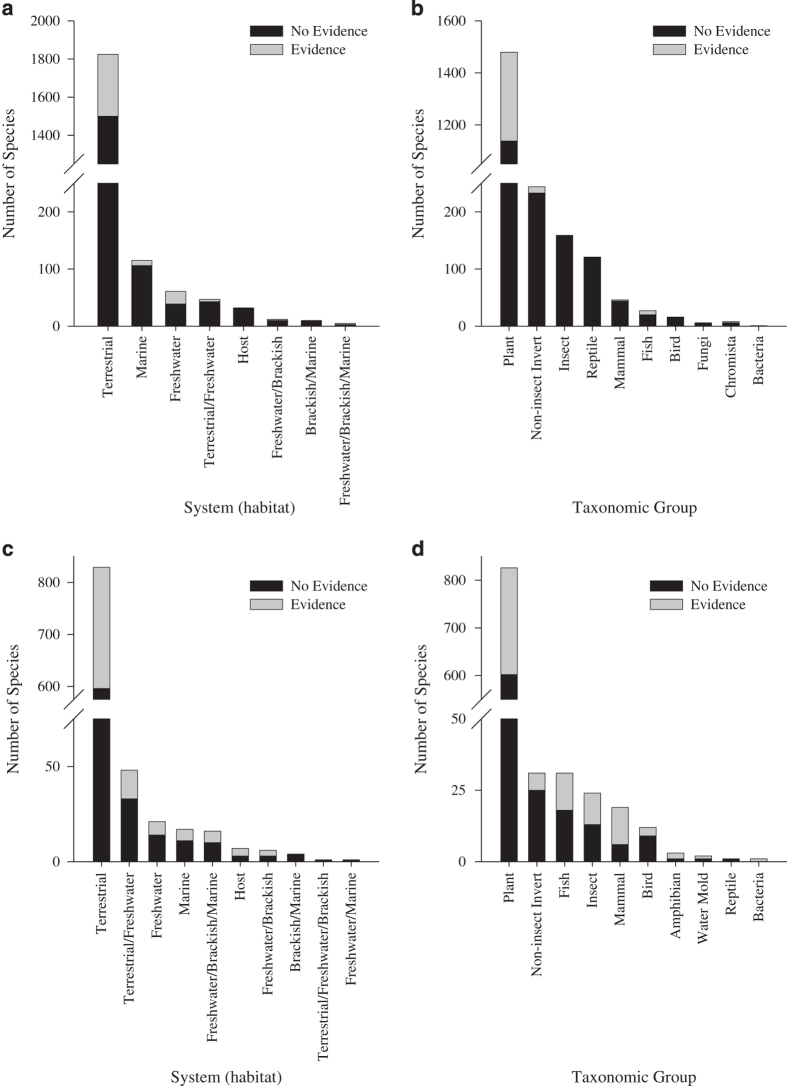
Example of data from GRIIS Checklists for individual countries. Number of alien species recorded (**a**,**b**) in South Africa (S=2,108) and (**c**,**d**) in Chile (S=950, including Juan Fernandez and Easter Islands) by (**a**,**c**) environment and (**b**,**d**) taxonomic group. Grey bars represent species with evidence of impact (Table 2, Fig. 2). Black bars indicate species with no evidence of impact. Note that ‘no evidence of impact’ does not mean that a species does not have any impact, rather that there is currently no evidence that it does.

Across the 20 countries used as exemplars for this Data Descriptor (Data Citation 1 to Data Citation 23), there are currently 6,414 species listed and 11,320 records. The number of species in these exemplar Checklists ranged from 77 (Mongolia) to 2107 (South Africa) (Table 3). To illustrate the data records, the checklists from South Africa (an example of a country where marine taxa are well represented) and Chile (an example of a country including island checklists) are used to show the distribution of species across systems and higher taxa, and the balance of species for which there is or is not evidence of impact (Fig. 3). Table 3 summarises the information in these exemplar checklists.

**Figure 4:**
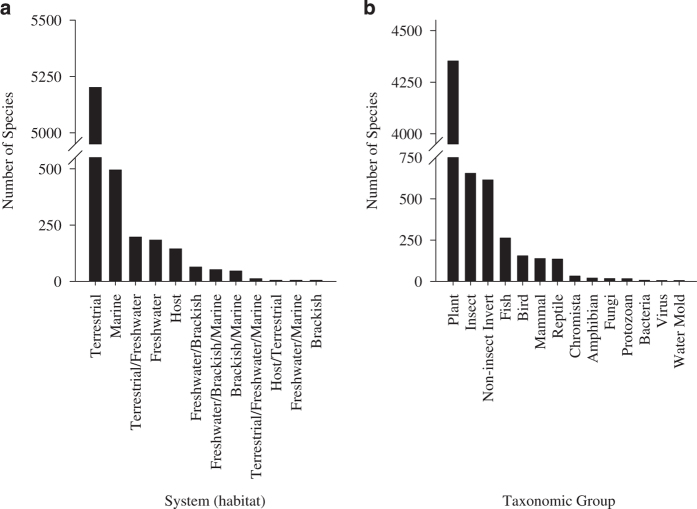
Example of how GRIIS may be used for global reporting on the state of invasion by species that impact biodiversity and ecosystems. Data collated across the 20, exemplar country GRIIS Checklists (including three sub-lists of islands associated with national mainlands) as examples of the use of these data for global reporting. Number of alien species (s=6,416) recorded for the pooled 20 exemplar countries by (**a**) system (habitat) and (**b**) taxonomic group.

**Figure 5:**
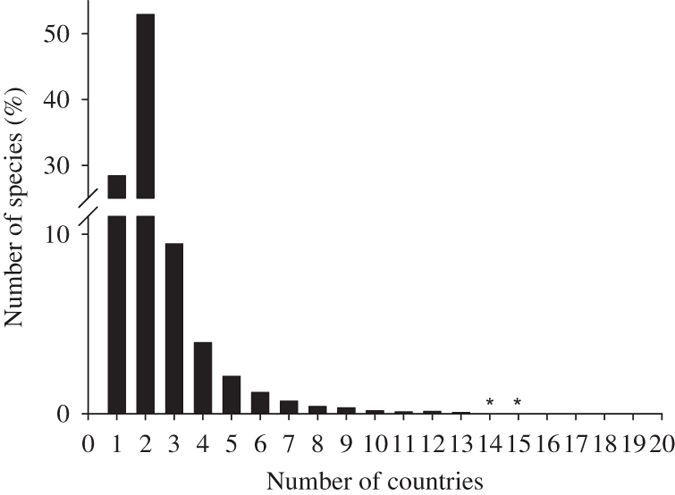
Frequency of species across the 20 exemplar countries. The percentage of species present in only one, two or three countries up to the number of species (2 in this case) present in the maximum number of countries for any species (*).

Beyond country use, these data will be used for reporting on the global status of biological invasion and to support indicators of biological invasion^13^. For example, collating the information across checklists shows the distribution of species across systems and higher taxa (Fig. 4). The frequency of particular species across countries provides information for prioritising invasive species based on the extent of their global distributions (Fig. 5). Of the 6,414 species across the 20 countries, more than 80% had evidence of impact in at least one to two countries (Fig. 5).

### Known data gaps and uncertainties

There are a number of common sources of misinterpretation associated with alien and invasive species databases and the site or taxon-based lists that they encompass. First, there is often lack of clarity on the scope, definitions and protocols used to design, structure and populate alien and invasive species databases. One of the principal objectives of this Data Descriptor is to clearly describe and define the scope, data and information types in GRIIS, to facilitate appropriate interpretation and use of GRIIS.

Second, alien and invasive species databases and lists are by nature dynamic for multiple reasons, including the population and range dynamics of alien species, the time lag that often occurs between establishment and spread of alien species outside of their native ranges, and time lags between such events and the quantification and documentation of their impacts^15^. Additionally, species checklists may be prescribed by national regulatory or other governance-based criteria that differ from the outcomes of scientific risk analysis. Other biases include those well known for alien species including geographic bias and taxonomic gas, language barriers to information access, and inconsistent use of invasion terminology across information sources^15^.

No alien and invasive species list or database is accurate or complete and should rather be regarded and appreciated, at least in the case of GRIIS, as an evidence-based information repository designed for the purpose of improving and monitoring change in alien and invasive status and properties. A shortage of country taxon specific editors to review draft checklists is a recurring problem, as well as response times for the reviews.

Nonetheless, by providing a transparent, traceable process of data collation, GRIIS provides a robust basis for delivering information on alien and invasive species needed by countries, as well as for global reporting, a baseline for ongoing updates, and a platform for sustainable delivery of this information. Future updates of the country lists in GRIIS will occur as a result of (i) improved knowledge of alien taxa and their impacts in countries, and (ii) the dynamics of alien and invasive species distributions as a consequence of ongoing range expansions as well as reductions as a consequence of control efforts.

## Data Records

GRIIS is based on PostgreSQL, an open source object-relational database system (PostgreSQL Global Development Group 2017; License: PostgreSQL License (https://opensource.org/licenses/postgresql). The compiled data are accessible via two avenues, the GRIIS Homepage (http://www.griis.org/) and from GBIF.org (https://www.gbif.org/), and we make a distinction here between these two knowledge products of the GRIIS resource:

1. The GRIIS Homepage is a public interface that is readily accessible to a wide range of users for *ad hoc* use and download. The GRIIS Homepage provides an interface that allows both basic and advanced searches and downloads for 198 countries. Basic searches include queries using species names. Other search options include by country, by kingdom, by environment/system, species whose status and impacts in a specific country have been verified.
2. The GRIIS Checklists of Introduced and Invasive Species for each country are available for research or official use via GBIF.org. GRIIS Checklists are published through an installation of the Integrated Publishing Toolkit (www.gbif.org/ipt) managed by the Atlas of Living Australia (ALA, https://www.ala.org.au/), to become accessible via GBIF.org, the web portal of the Global Biodiversity Information Facility (GBIF). Checklist publication through GBIF.org allows for their integration with the GBIF.org Backbone Taxonomy as well as subsequent filtering of the global occurrence records by those checklists.

For each species in each country, GRIIS provides five categories of information: taxonomy; habitat; occurrence and origin status; evidence of impact; and updates and source information (Table 2). There are 10 fields visible on the results page of the GRIIS Homepage with additional information on species synonyms visible via a clickable information link in the ‘Name’ field (Table 2). In the GRIIS Checklists in GBIF.org there are 15 related fields of information for each country, with terms following the Darwin Core standard (DwC)^20^ (http://rs.tdwg.org/dwc/terms). Table 2 describes the mapping of the key GRIIS data components, including terms used on the GRIIS Homepage and within the GRIIS Checklists in GBIF.org.

For data to be published through the IPT of GBIF.org, the data need to conform to DwC. DwC is a body of standards developed to facilitate sharing of biodiversity related information. It is primarily based on taxa and their documented occurrence in nature^20^. DwC terms can also be extended and adapted using two additional relevant extensions of the standard, i.e., ‘species distribution’ and ‘species profile’ (https://tools.gbif.org/dwca-validator/extensions.do). In addition, checklists through GBIF.org come with comprehensive metadata associated with each dataset.

## Technical Validation

Once the draft checklists were prepared, the data were re-checked to validate all records. As described above, validation involves the harmonisation of taxonomy, assigning environment/system for each species, checking that the interpretation of the taxonomic status of the species, its origin, occurrence status and evidence of impact (Tables 1 and 2), are correctly recorded.

## Usage Notes

GRIIS’s principal focus is on naturalised taxa within countries, for which there is evidence of environmental impact there or elsewhere. The purpose is to assist countries to prioritize interventions, manage, monitor and report on the state of biological invasion in their countries. GRIIS provides countries with a resource for managing, updating, prioritising and communicating invasive species information, including contributing to progress towards achieving Aichi Target 9^21^.

GRIIS also provides information for use by the broad range of sectors relevant to and interested in biological invasions (conservation agencies, local governments, industry and researchers). For example, GRIIS Checklist publication through GBIF.org allow for their integration with GBIF.org Backbone Taxonomy and subsequent filtering of species occurrence records by those checklists.

GRIIS provides a systematic, readily accessible, globally representative, policy-relevant platform of information on biological invasions, especially for national and global reporting on trends in biological invasion. The ultimate goal of GRIIS is therefore to establish and provide sustainable national and global baselines from which to monitor changes in the status of alien and invasive species.

## Additional Information

**How to cite this article:** Pagad, S. *et al.* Introducing the Global Register of Introduced and Invasive Species. *Sci. Data* 5:170202 doi: 10.1038/sdata.2017.202 (2018).

**Publisher’s note:** Springer Nature remains neutral with regard to jurisdictional claims in published maps and institutional affiliations.

## Supplementary Material



## Figures and Tables

**Table 1:** Glossary of terms used here and in the Global Register of Introduced and Invasive Species.

**Term used in GRIIS**	**Description/definition**
Alien (synonymous with Introduced)	A species, subspecies or (for plants) variety or cultivar, moved by human activities beyond the limits of its native geographic range, or resulting from breeding or hybridization and being released into an area in which it does not naturally occur^16^, and includes any part, gametes or propagule of such species that might survive and subsequently reproduce.
Cryptogenic	Species of unknown biogeographic history that cannot be ascribed to being native or alien (modified from^22^).
Uncertain	Species recognised as clearly alien although their specific geographic origin is unknown^22^.
Data products	In this case specifically the GRIIS Homepage, and the GRIIS Checklists for countries.
Introduced	See Alien
Invasive	A taxon whose introduction and/or spread threatens biological diversity (Convention on Biological Diversity; http://www.cbd.int/decision/cop/).
Checklist	A regional, national or thematic taxonomic enumeration.
Inventory	A comprehensive list of species, usually relating to a specific survey event.
Native-Alien	Species native to some areas of a country or territory but introduced by humans into places outside of their natural range of distribution in that country, where they become established and disperse.
Naturalised (synonymous with Established)	Those alien species that sustain self-replacing populations^22^.
Origin (synonymous with Provenance)	The area in which a species arose and/or where it first arrived by natural means (through range expansion), without human intervention (modified from^22^).

**Table 2:** Matching of data fields and terms used on the Global Register of Introduced and Invasive Species (GRIIS) Homepage with those used in GRIIS Checklists (for Darwin Core term definitions see http://rs.tdwg.org/dwc/terms).

**GRIIS Homepage field terms**	**Related Darwin Core standard term/s used in country GRIIS Checklists**	**Description of GRIIS terms listed under the five main information categories**
		**1. Taxonomy**
Name	taxonID	*Species scientific name and species authority* as listed in the source and accepted scientific name (if different). The Global Biodiversity Information Facility (GBIF) Backbone Taxonomy is used as the taxonomic editor to harmonise species scientific names across all country GRIIS Checklists. The species scientific name as recorded in the primary information source is listed; if the species scientific name is listed as a synonym in GBIF, the accepted name is listed together with the species synonym. GRIIS splits the scientific name and species authority into two fields.
Authority	scientificName	
	acceptedNameUsage taxonRank taxonomicStatus	
Synonyms used in the country		On the GRIIS Homepage the ‘Accepted scientific name’ is included in the primary list and the synonym used by the source information can be viewed as a comment. In the GBIF view, if a synonym is used by the source information, the accepted name is listed under acceptedNameUsage and the synonym is included in the scientific ‘Name’ field.
Kingdom	kingdom	Higher taxonomy, sourced from the GBIF Backbone Taxonomy. The GRIIS Homepage includes only Kingdom as part of the higher taxonomy of a species. The GRIIS Checklists include kingdom, phylum, class, order, and family.
	phylum	
	class	
	order	
	family	
		**2. Habitat**
System [***Terms in field**: marine; freshwater; brackish; terrestrial; host; and relevant combinations of these*]	realm habitat associatedTaxa	The environment in which the species occurs or that it is associated with. This could include a host plant or host animal in the case of parasites or plant diseases, for example.
		**3. Occurrence and origin status**
Origin [***Terms in field**: Alien; Native/Alien; Cryptogenic/Uncertain*]	occurrenceStatus establishmentMeans	Origin (provenance) of the species. Three options have been considered in the classification of the non-native origin: Alien (species that have been introduced outside their natural range by human action); Cryptogenic/ Uncertain (species whose origins are uncertain); Native-Alien (species that are native to one area of the country and alien and invasive in another).
Country	countryCode	Country or territory where the species occurs. This includes all countries that are Party to the Convention on Biological Diversity (CBD), and EU island territories and dependencies. The list of country names includes the list of Parties to the CBD and the list of EU island territories and dependencies as listed by the European Union (UN member states and EU Overseas Countries and Territories).
Verified [***Terms in field**: yes*]		Verification of the origin, occurrence and impact of the alien species within the country by nominated Country Editors.
		**4. Impact**
Impact [***Terms in field**: yes; no*]	isInvasive	Evidence of impact—A ‘yes’ for evidence of impact denotes that the alien species is ‘invasive’ in that country. A systematic decision-making process was used to decide if a species was classified as ‘invasive’ at a country level (see Fig. 2).
		**5. Updates and sources**
Date	versions	The last modified date of a species record is recorded. A version number is assigned to every version of the GRIIS Checklist published through the IPT, at the conclusion of an annual update (for e.g., ver1, ver2). All incremental updates within a calendar year will result in a subsidiary number being assigned to the current version (e.g., ver2.1, ver3.2).
Source		Complete list of the sources of information/references for every species record. Also included are sources of information and references for every species record and an indication if the species record has been verified by a Country Editor. Webpages with GRIIS Checklists published through GBIF.org display recommended DOI-enabled citation constructed from the checklist metadata elements.
Terms are listed here by information category and not in the same order in which they appear in the data products.		

**Table 3:** Summary information for GRIIS data in 20 country Checklists and three sub-lists.

**Country**	**ISO Code**	**Region**	**Data citation #**	**Total species in this checklist (n)**	**Kingdom Animalia %**	**Kingdom Plantae %**	**Other Kingdoms %**	**Terrestrial %**	**Freshwater %**	**Brackish %**	**Marine %**	**Host %**	**Invasive %**
Brunei Darussalam	BN	Asia-Pacific	1	133	17	83	0	86	17	2	2	0	5
Chile	CL	Latin American and Caribbean	2	844	14	86	1	91	11	3	5	0	29
Chile- Juan Fernandez Islands	CL	Latin American and Caribbean	3	120	14	86	0	100	0	0	0	0	17
Chile- Rapa Nui- Easter Islands	CL	Latin American and Caribbean	4	121	6	94	0	100	1	0	0	0	30
Cook Islands	CK	Asia-Pacific	5	469	8	92	0	97	5	1	0	1	36
Cuba	CU	Latin American and Caribbean	6	692	16	83	1	92	10	1	1	1	55
Croatia	HR	Eastern European	7	899	26	72	2	79	7	3	11	6	12
Egypt	EG	Africa	8	444	55	42	2	43	13	6	48	1	10
Ireland	IE	Western European	9	1266	32	66	2	89	5	1	6	2	5
Mongolia	MN	Asia-Pacific	10	77	55	45	0	79	21	5	0	4	4
Myanmar	MM	Asia-Pacific	11	162	25	72	2	77	25	6	4	3	41
Nepal	NP	Asia-Pacific	12	245	25	75	0	85	16	3	0	3	18
Russian Federation	RU	Eastern European	13	1297	26	74	1	87	11	6	6	2	19
Saint Lucia	LC	Latin American and Caribbean	14	113	41	59	0	91	13	3	1	2	33
Saudi Arabia	SA	Asia-Pacific	15	159	47	52	1	78	20	10	13	3	18
Seychelles	SC	Asia-Pacific	16	966	5	95	0	99	2	0	0	0	11
South Africa	ZA	Africa	17	2107	29	70	1	89	6	1	6	2	17
Sri Lanka	LK	Asia-Pacific	18	184	38	62	1	72	33	9	3	1	26
Tunisia	TN	Africa	19	152	62	35	3	38	14	10	47	6	41
United Arab Emirates	AE	Asia-Pacific	20	247	86	2	12	89	26	5	10	2	26
Vanuatu	VU	Asia-Pacific	21	264	25	75	0	94	6	0	4	0	35
Yemen	YE	Asia-Pacific	22	233	11	89	0	98	3	0	0	0	9
Yemen- Soqotra	YE	Asia-Pacific	23	135	22	78	0	99	4	1	1	0	18
Habitat types are combinations of the four main systems considered, i.e., terrestrial, freshwater, brackish, marine, as well as biological hosts of introduced species. The percentage of species in each checklist that have country (island)-level evidence of impact (i.e. are invasive) is also provided. #Full data citations are provided below.													
